# Expect more from us…

**DOI:** 10.4103/0019-5545.44896

**Published:** 2009

**Authors:** T. S. Sathyanarayana Rao

**Affiliations:** Department of Psychiatry, JSS University, JSS Medical College Hospital, Ramanuja Road, Mysore - 570 004, India. E-mail: tssrao19@yahoo.com

“Start by doing what is necessary

Then do what is possible.

And suddenly, you are doing the impossible.”

So goes a saying and has been our motto. Stepping into New Year, let me wish you all ‘A Very Happy New Year’ along with my team. It is also the time worth looking into what has been achieved and what you can look forward to in the coming year.

## SCHEDULE

All the issues of 2008, special supplement of January on Dementia and the January – March 2009 issue are already in our website and hard copies are being distributed to members at ANCIPS. The posting of copies will commence after ANCIPS 2009 conference. Possibly this is happening for the first time in the history of our journal. But this is a necessity rather than anything special because the above scheduling is essential if we are serious about getting the journal indexed. However, the general body has to look into this issue next year in view of elections due then. As the new editor needs time to settle, if changes do come about, a provision has to be made to the publication of the Jan-march issue by the previous editor himself, preferably before ANCIPS. I seek the permission of the esteemed EC and the members.

## ARCHIVING THE JOURNAL

I am very happy to present a DVD containing:

1. All the published issues of IJP since 1958-2008. A powerful search engine has been embedded to data mine based on titles, authors, abstracts and keywords. Many people have supplied the issues of the past journals but some names deserve a special mention. I am grateful to Dr. O. Somasundaram, who supplied majority of the issues, Dr. Chittaranjan Andrade from Bangalore and Dr. Smita N. Deshpande from New Delhi for supplying the journals from their private collections.
2. All the published issues of Indian Journal of Neurology & Psychiatry for the years 1949- 1953 edited by Dr. N.N. De and Dr. A.K. Deb respectively. It had stopped publication between 1954-1957 and it came out with the current name of Indian Journal of Psychiatry in 1958. I am grateful to Dr. Haque Nizamie, Director & Dr. D. Ram, Professor, Central Institute of Psychiatry, Ranchi for all the interest and effort in getting these copies scanned and transmitted to us for processing. The Commemorative Volume featuring 50 golden years was published in January 2008 and for those still missing the issue and interested in it, it is available as a supplement at our website.
3. As a service to the society, we have also included the clinical practice guidelines edited and published by Dr. Shiv Gautham & Dr. Ajit Avasthi for the Indian Psychiatric Society. It contains:
  - First Publication of 2004 covering 6 major disorders – Schizophrenia, Depression, Bipolar disorders, Obsessive compulsive disorders, Generalized anxiety disorder and panic disorder
  - Second Edition covering - substance abuse disorders, sexual dysfunctions & sleep disorders (2005)
  - Third Edition covering – Geriatric Psychiatry (2006)
  - Fourth Edition covering – Child Psychiatry (2007)
    I am grateful to Dr. Shiv Gautham & Dr. Ajit Avasthi for all the help and effort in getting the CPGs.
4. We have also included free the photo-directory containing the updated membership codes, names, addresses and photos of all the members in PDF format. I must thank our treasurer Dr. Asim Kumar Mallick from Kolkata and his team for all the effort and help.

The preparation of the DVD was a mammoth task and I will be failing in my duty if I do not thank the whole team that put up their heart and soul in the task. I must thank in addition to Dr. O. Somasundaram, from Chennai, the PGI group led by Dr. P. Kulhara, Dr. Ajit Avasthi and Dr. Sandeep grover for the input which changed our archiving ways dealing with digitalization. Dr. Swaminath G. & Dr. Ajit Bhide have spent many hours and days to beat the deadline. My thanks to the PGs in our department - Dr. Abhijith H., Dr. Kiran Kumar K, Dr. Smitha T., Dr. Nimisha Mishra and Dr. Saraswathi N. I also thank the other staff in the department - Dr. Dushad Ram, Asst. Professor, Dr. L. Sam S. Manickam, local coordinator, Dr. VST Krishna, PSW and Prof. P. Kalaiah, Dr. Rajesh Raman, Associate Professor and Dr. Bindu. A for their active involvement. My thanks to Mrs. Renuka B.S of Vijaya foundation, Mysore and Roopa N. from Bangalore for review and editing. My thanks are also due for Dr. Ajit Bhide and Dr. Chittaranjan Andrade for reviewing the DVD. I am grateful to Mr. Madhushetty, CEO and Mr. Srinath and Mr. Raghu of Indigo Informatics at Bangalore for the excellent job executed with aplomb. I also thank Mr. Vasudev Bhatt from Ramya creations, Mysore for the work and help all through. My sincere thanks to M/s. Sun Pharmaceuticals, Mumbai, who have sponsored it fully under a special educational grant and I am specifically grateful to Mr. Dilip Sanghvi, CEO, Mr. T.K. Roy, Senior vice president & Mr. K. Nanda Kumar, Vice President who personally took interest in the whole project.

## THE SPECIAL SUPPLEMENT ON DEMENTIA

I am very happy to present the special issue of Dementia, truly a collectors' volume to all the members. Dr. Shaji K.S from Trissur was the guest editor and all the credit must go to him for his very systematic effort in getting the invited and original articles numbering 21 in time and setting it in order. In view of the space constraint some of the articles and the original articles received for the supplement have appeared in this issue. The supplement is already available at our website and hard copies are available for distribution at ANCIPS 2009 at Agra. I am grateful to Mr. Vivek Seth, CEO of Intas Pharmaceuticals, Ahmedabad for sponsoring the issue fully. I also thank Mr. Ravi Prasad L., Sales Manager and Mr. Shivakumar T. M. of Intas Pharmaceuticals for their personal interest.

## THE YEAR 2009

I am confident the New Year will usher in many new changes so also many new faces. We intend to approach NHS for indexing purposes early this year. Dr. Mohan Das, incoming president has promised to lead the process. Many national and international academicians and researchers have promised their helping hand. Journal will have new sections like Psychiatric Pearls, the section under Dr. Vikram Yeragani and many more. Dr. Ajit Bhide is doing excellent service so also Dr. Vikram Patel, Dr. Chittaranjan Andrade and others. I am confident the new year will be a milestone year in the annals of IPS.

## EXPECT SOME MORE……

I have one dream fulfilled of what I had expressed in my first editorial – golden jubilee, 2008 of digitalizing and archiving of IJP. Luckily with every one contributing the DVD contains much more than that. For the future, as I know there are excellent publications available at Zonal, State and Local branch levels which are not accessible to many members and as such lost to the society. How about digitalizing all these publications? I am confident that it could be done by ANCIPS 2010! If society wills, budgetary constraint can be overcome one way or other.

